# Computed Tomography-Defined Fat Composition as a Prognostic Marker in Gastric Adenocarcinoma: A Systematic Review and Meta-Analysis

**DOI:** 10.1159/000527532

**Published:** 2022-10-13

**Authors:** Hans-Jonas Meyer, Andreas Wienke, Maciej Pech, Alexey Surov

**Affiliations:** ^a^Department of Diagnostic and Interventional Radiology, University of Leipzig, Leipzig, Germany; ^b^Institute of Medical Epidemiology, Biostatistics, And Informatics, Martin-Luther-University Halle-Wittenberg, Halle (Saale), Germany; ^c^Department of Radiology and Nuclear Medicine, University of Magdeburg, Magdeburg, Germany

**Keywords:** Meta-analysis, Systematic review, Gastric cancer, Visceral obesity

## Abstract

**Background:**

Computed tomography (CT)-defined fat quantification has been an emergent field of research in oncology. It was shown that this parameter is predictive and prognostic of several clinically relevant factors in several tumor entities.

**Objective:**

Our aim was to establish the effect of visceral (VFA) and subcutaneous fat areas (SFA) on overall survival (OS), disease-free survival (DFS), and postoperative complications in gastric cancer patients based on a large patient sample.

**Methods:**

MEDLINE library, EMBASE, and SCOPUS databases were screened for the associations between VFA and SFA defined by CT images and OS, DFS, and postoperative complications in gastric cancer patients up to August 2022. The primary endpoint of the systematic review was the hazard ratio for the outcome parameters. High VFA was, in most studies, defined by the threshold value of 100 cm<sup>2</sup>. In total, 9 studies were suitable for the analysis and included in the present study.

**Results:**

The included studies comprised 3,713 patients. The identified frequency of visceral obesity was 44.9%. The pooled hazard ratio for the effect of high VFA on OS was 1.28 (95% CI 1.09–1.49, *p* = 0.002). For SFA, it was 1.87 (95% CI 1.45–2.42, *p* < 0.0001). The pooled hazard ratio for the influence of high VFA on DFS was 1.17 (95% CI 0.95–1.43, *p* = 0.14). The pooled odds ratio for the associations between VFA and postoperative complications was 1.36 (95% CI 1.09–1.69, *p* = 0.006).

**Conclusion:**

CT-defined VFA and SFA influence OS in patients with gastric cancer. VFA also influences the occurrence of postoperative complications. Therefore, assessment of fat areas should be included in clinical routine in patients with gastric cancer.

## Introduction

Body composition is an emergent research field. The main purpose of investigations is the characterization and quantification of fat and muscle constitution of the body [1, 2, 3, 4, 5, 6, 7]. It can be defined by cross-sectional imaging, which is proposed to be a reliable method to quantify the skeletal muscle and different fat areas [1, 2, 3, 4, 5, 6, 7]. There is growing literature on the predictive and prognostic relevance of these parameters. In most studies, these areas are calculated on computed tomography (CT) images. One CT slice is used of the L3 intervertebral height to measure subcutaneous and visceral fat areas (SFA and VFA) [4]. This is especially interesting in oncologic patients as CT imaging is performed for staging purposes for initial diagnosis and follow-up investigations.

Gastric cancer patients are at great risk of cachexia and sarcopenia [8, 9, 10]. As a first reason, most patients are in advanced tumor stages with a greater risk for cachexia. As a second reason, possible treatment options like gastrectomy and systemic treatment are associated with malnutrition and consequently risk for sarcopenia [10]. This was shown in preliminary analyses that low skeletal muscle mass and VFAs are associated with mortality and postoperative complications in gastric cancer patients, emphasizing the importance of body composition in this tumor entity [10]. However, there is a lack of systematic data regarding the associations between body composition parameters and clinical outcome parameters in gastric cancer patients based upon a meta-analysis design. Therefore, the purpose of the present systematic review and meta-analysis was to identify the associations between fat areas defined by CT images with overall and disease-free survival as well as postoperative complications in gastric cancer patients.

## Patients and Methods

### Search Strategy

MEDLINE library, EMBASE, and SCOPUS databases were screened for papers regarding low skeletal muscle mass evaluation in gastric cancer patients up to August 2022. The Preferred Reporting Items for Systematic Reviews and Meta-Analyses (PRISMA) statement was used for the literature acquisition [11]. The PRISMA checklist is provided within the supplementary material.

**Fig. 1:**
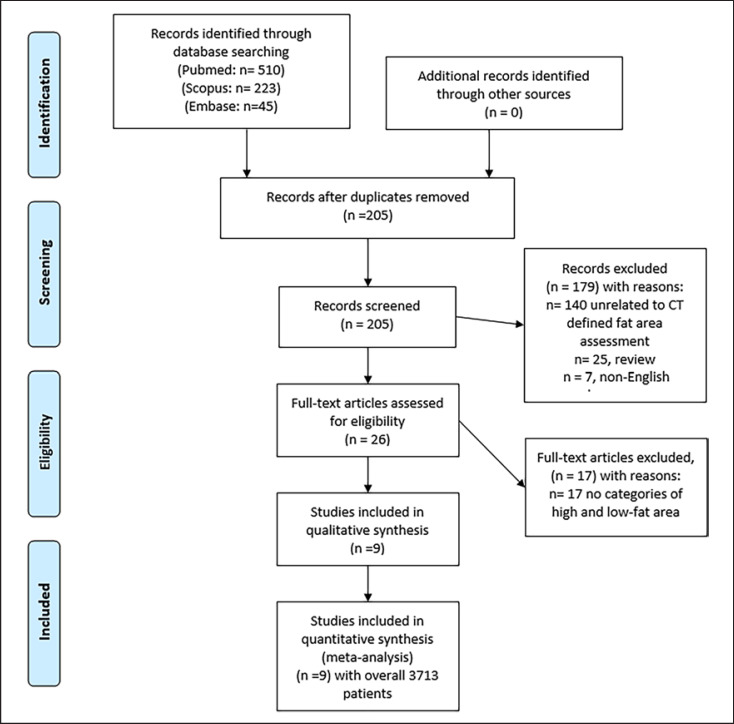
PRISMA flowchart provides an overview of the paper acquisition. Overall, 9 studies with 3,712 patients with gastric cancers were included in the analysis.

The paper acquisition is summarized in Figure 1. After a thorough review, 9 studies were suitable for the present analysis [12, 13, 14, 15, 16, 17, 18, 19, 20]. The following search words were used: “gastric cancer” OR “gastric carcinoma” AND “body composition” OR “visceral fat” OR “visceral fat area.”

The primary end point of the systematic review was the hazard ratio for CT-defined fat areas with a reported confidence interval. The hazard ratio was defined as high versus low fat area, as reported by the included study.

Inclusion criteria: studies (or subsets of studies) were included if they satisfied the following criteria: (1) gastric cancer patients, (2) fat areas defined by CT, and (3) reported odds ratio or hazard ratio with standard deviation. Exclusion criteria were (1) systematic reviews, (2) case reports, (3) non-English language, and (4) fat areas defined on other modalities than CT.

### Data Extraction

Data extraction was performed by H.J.M. followed by an independent evaluation of extractions for correctness by A.S. For each study, details regarding study design, year of publication, country of origin, patient number, mean patient age, diagnosis and tumor stage, treatment, fat area definition, threshold values, overall survival (OS) outcome results, and adjustment factors were extracted.

### Quality Assessment

The quality of the included studies was assessed by the Newcastle-Ottawa Scale (http://www.ohri.ca/programs/clinical_epidemiology/oxford.htm) [21]. Study quality assessment was conducted by two authors (HJM and AS) and mainly included the selection of cases, comparability of the cohort, and outcome assessment of exposure to risks. A score of 0–9 was assigned to each study, and a study with score ≥6 was considered to be of high quality.

### Statistical Analysis

The meta-analysis was performed using RevMan 5.3 (2014; Cochrane Collaboration, Copenhagen, Denmark). Heterogeneity was calculated by means of the inconsistency index *I*^2^ [22, 23]. DerSimonian and Laird random-effect models with inverse-variance weights were performed without any further correction [24].

## Results

### Quality of the Included Studies

Table 1 gives an overview of the included studies. 3 studies (33.3%) were of retrospective design, and 6 studies (66.7%) were of prospective design. The overall risk of bias can be considered low to moderate, indicated by the high Newcastle-Ottawa Scale values throughout the studies (Table 2).

### Patients

The included studies comprised overall 3,713 patients, 976 female (26.3%), with a mean age of 62.8 years, ranging from 55 to 72 years. Most studies (*n* = 7, 77.8%) were based in Asia. Two studies (22.2%) reported patients from South America, Brazil [12, 13].

Two studies included other cancers in addition to gastric cancer patients. Bitencourt et al. [12] also included esophageal-gastric junction cancer, and Carvalho et al. [13] also included colorectal cancer patients in the analysis [12, 13]. All cases were histopathologically defined as adenocarcinomas. All studies investigated patients undergoing surgical treatment, most often radical gastrectomy. For detailed information, see Table 1.

### Fat Area Assessment

All CT images were acquired with 5-mm slice thickness or less. Various methods were reported to measure the fat areas. In seven studies, the VFA was measured in a single CT slide using the VFA [12, 13, 14, 15, 16, 18, 19]. Out of these, the level of the CT slide was at L3 in five studies [13, 14, 15, 16, 19], at L4/5 in one study [12], and at umbilical cord level in another study [18]. In addition, the subcutaneous fat area was assessed, and the SFA was calculated in four studies [12, 14, 15, 16]. Finally, in two studies, an index approach was performed [17, 20]. In detail, the visceral fat index (VFI) and the subcutaneous fat index were calculated, which were set in relation to the height of the patient [17, 20]. Dong et al. [15] and Huang et al. [16] calculated the VSR, which represents the ratio of VFA to SFA [15, 16]. Different threshold values were used to determine visceral obesity. The threshold value of 100 cm^2^ was used in 6 studies [12, 14, 15, 16, 18, 19]. In one study, a threshold of 163.8 cm^2^ was used for men, and a threshold of 80.1 cm^2^ was used for women [13]. A threshold >40.8 cm^2^/m^2^ was reported for the VFI in a single study [17]. Finally, the thresholds for the VSR were reported to be 1.33 in males and 0.93 in female patients [20].

### Frequency of High Visceral Fat Area

The proportion of patients with high VFA, termed visceral obesity, ranged from 35.9% to 53.7%. The calculated mean proportion was 44.9%. A high heterogeneity was identified between the studies. 2 studies [14, 16] did not report the frequency of high VFA in the studies.

### Overall Survival

**Fig. 2:**
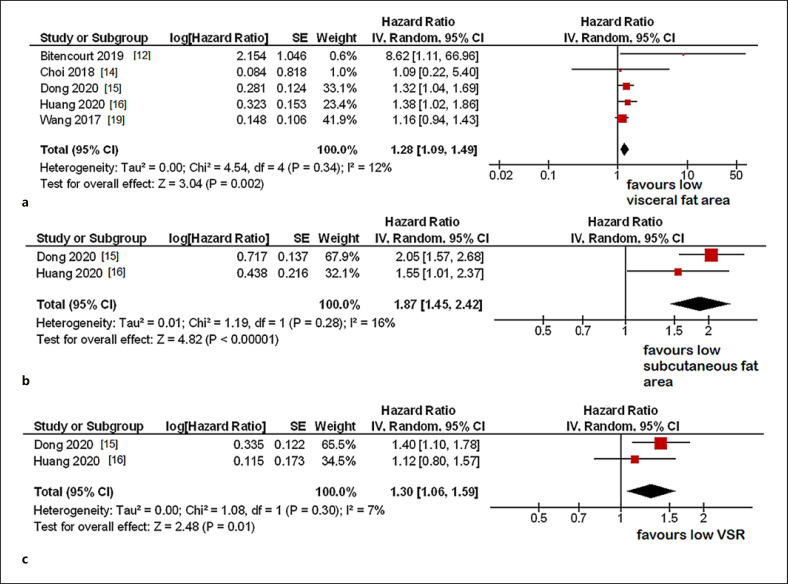
**a** Forrest plots of the effect of high VFA on OS in univariable analysis. The calculated hazard ratio was 1.28 (95% CI 1.09–1.49). **b** Forrest plots of the effect of high SFA on OS. The pooled hazard ratio for the associations between SFA and OS in univariable analysis was 1.87 (95% CI 1.45–2.42). **c** Forrest plots of the effect of high VSR on OS. The pooled hazard ratio was 1.30 (95% CI 1.06–1.59).

In 5 studies with 2,769 patients, relationships between OS and VFA were investigated. The pooled hazard ratio for the associations between VFA and OS in univariable analysis was 1.28 (95% CI 1.09–1.49, *p* = 0.002) (Fig. 2a). The heterogeneity among the studies was low (*I*^2^ = 12%). Two studies with 1,744 patients analyzed SFA [14, 16]. The pooled hazard ratio for the associations between SFA and OS in univariable analysis was 1.87 (95% CI 1.45–2.42, *p* < 0.0001). Also, for the studies, the heterogeneity was low (*I*^2^ = 16%) (Fig. 2b). The same two studies were included in the analysis between VSR and OS [14, 16]. The pooled hazard ratio for the associations between SFA and OS in univariable analysis was 1.30 (95% CI 1.06–1.59, *p* = 0.01), with heterogeneity of 7% (Fig. 2c).

### Disease-Free Survival

**Fig. 3:**
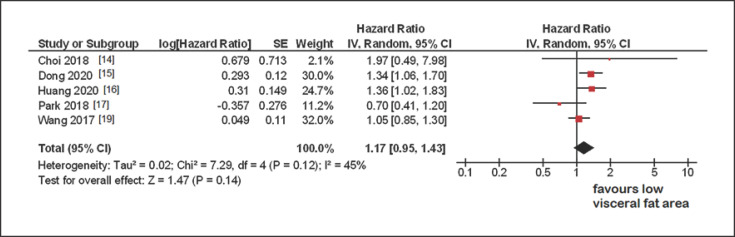
Forrest plots of the effect of high VFA on DFS. The pooled hazard ratio was 1.17 (95% CI 0.95–1.43).

In 5 studies with 2,835 patients, associations between disease-free survival and VFA were analyzed. The pooled hazard ratio in univariable analysis was 1.17 (95% CI 0.95–1.43, *p* = 0.14) (Fig. 3). The heterogeneity across the studies was moderate (*I*^2^ = 45%).

### Postoperative Complications

**Fig. 4:**
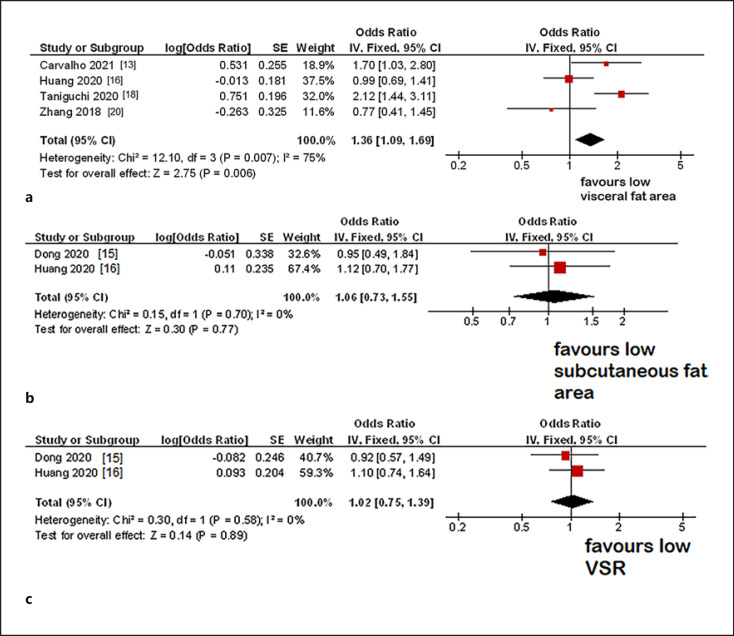
**a** Forrest plots of the effect of high VFA on postoperative complications. The pooled odds ratio was 1.36 (95% CI 1.09–1.69). **b** Forrest plots of the effect of high SFA on postoperative complications. The pooled odds ratio was 1.06 (95% CI 0.73–1.55). **c** Forrest plots of the effect of high VSR on postoperative complications. The pooled odds ratio was 1.02 (95% CI 0.75–1.39).

Overall, 4 studies with 1,405 patients were included in the analysis between VFA and postoperative complications. The pooled odds ratio for VFA in univariable analysis was 1.36 (95% CI 1.09–1.69, *p* = 0.006) (Fig. 4a). The heterogeneity among the studies was high (*I*^2^ = 75%). In 2 studies with 1,744 patients, the influence of VSR and SFA on the occurrence of postoperative complications was investigated [14, 16].

The pooled odds ratio for SFA was 1.06 (95% CI 0.73–1.55, *p* = 0.77) (Fig. 4b), and for VSR, it was 1.02 (95% CI 0.75–1.39, *p* = 0.89) (Fig. 4c). There was no heterogeneity across the studies (*I*^2^ = 0%).

## Discussion

The present analysis identified a significant association of VFA and SFA with OS, which highlights the importance of assessment of body composition in gastric cancer. Interestingly, only VFA was associated with the occurrence of postoperative complications, whereas SFA and VSR were not. A key finding can be summarized that VFA might be a more valuable parameter in clinical routine. The topic of body composition is an ever-growing research field with many clinically important applications and novel prognostic implications throughout almost every field of medicine [1, 2, 3, 4, 5, 6, 7, 8, 9, 10].

Especially, oncological patients are at risk of changes of body composition, comprising sarcopenia and cachexia for several reasons. At first, the cancer itself can cause muscle wasting and cachexia. Then there is the possible side-effect of the cancer treatment due to gastrectomy and systemic chemotherapy [19, 20]. That is why body composition assessment is of great importance in gastric cancer patients to provide new biomarkers. This is influenced by several mechanisms and several adverse events, comprising fatigue, loss of energy, and emotional distress [19, 20].

Early on, it was acknowledged that not only the muscle mass is of importance but also the amount of visceral adipose tissue. An important finding was the combination of sarcopenia and visceral obesity, which was shown to be a risk in several tumor entities [25, 26, 27].

The present analysis used defined groups stratified by cut-off values into high and low-fat areas. This approach is commonly used in the literature to easily stratify patients and to identify patients at risk for hazardous outcome.

Yet, contrary to the skeletal muscle assessment for sarcopenia, the definitions for high VFA are not as standardized. In most studies, a threshold value of 100 cm^2^ is employed [28]. Beyond that, there is no consent on whether an index parameter might be superior to the area measurement alone.

Regarding body composition assessment in gastric cancer patients, the first promising results were published in the literature [8, 9, 10, 29]. Thus, for sarcopenia defined as low-skeletal muscle mass, patients with preoperative sarcopenia had an increased risk of total postoperative complications (OR = 2.17, 95% CI = 1.53–3.08), severe complications (OR = 1.65, 95% CI = 1.09–2.50), and poorer OS (HR = 1.70, 95% CI = 1.45–1.99) in a meta-analysis based on 4,262 patients [29]. In a recent study on 840 patients, especially patients with sarcopenic obesity had worse outcome after gastrectomy (HR = 2.6, 95% CI = 1.313–5.179) [30].

However, there are also contrary results presented by a large-multicenter study investigating 761 esophageal-gastric junction cancers undergoing palliative chemotherapy [31]. A linear model of body composition parameter was employed in this study, and the patient sample was not stratified according to high and low fat areas [31]. Contrary to the present study, no association between VFA and SFA with OS was identified.

Established factors for long-term survival after surgery for patients with gastric cancer are affected by both oncological and nutritional associated factors. Moreover, baseline conditions of the patients, such as age and comorbidities, TNM stage, and differentiation of tumor, were identified to influence the long-term prognosis after surgery [15]. Presumably, assessment of body composition can provide novel and independent biomarkers to the existing clinical ones.

One concern of the present analysis is the identified heterogeneities, which are possibly caused by different patient samples in different tumor stages and various treatment options pooled together. However, for associations between SFA and VFA with OS in gastric cancer either none or low heterogeneity was observed in the present analysis.

Moreover, it should be acknowledged that different approaches to calculate the fat areas were employed. The acquisition level was predominantly at L3 with a fat area calculation on one slide. Only 2 studies used an index approach by dividing the VFA by the height to address possible confounding by the body height.

One potential concern of the reliability of the body composition parameters is that possible confounders can be different slice thickness inducing partial volume effects. However, there is enough data that single slice quantification of fat areas, both for MRI and CT, can be considered reliable with a high interreader agreement [32, 33, 34].

Nowadays, the segmentation of fat areas is semiquantitatively performed with resulting time consumption. Due to the advent of machine learning algorithm, the segmentation of fat areas will be performed automatically, which was already shown in a promising study based on 840 gastric cancer patients [30].

Another key finding was that only VFA was associated with postoperative complications, whereas SFA did not. This could lead to the association between VFA and OS in these patients but also have a direct association with the intraoperative conditions caused by visceral obesity. In a recent meta-analysis, visceral obesity was associated with an increased surgical site infection, pneumonia, and postoperative pancreatic fistula. Yet, the authors concluded that further studies are needed to better understand the complex interactions between postoperative complications and visceral obesity [28].

In gastric cancer patients, the assessment of fat areas, especially VFA has been highlighted as an important factor [35, 36]. So, it could aid to stratify patient groups undergoing surgical treatment [35] and could help to identify patients with limitation of chemotherapy treatment [36]. In short, every aspect of gastric cancer treatment could be guided by fat area assessment.

Pathophysiologically, VFA is associated with increased serum levels of inflammatory cytokines and angiogenic factors, which could promote tumor growth [37], which could influence the OS. Moreover, a higher intraperitoneal fat tissue could promote invasion and peritoneal metastasis of gastric cancer [38]. As another point, VFA has higher hormonal and metabolic activities than SFA [39, 40, 41]. Visceral adipocyte-induced insulin-like growth factor, inflammatory adipokines like interleukin-6 and tumor necrosis factor-α, and angiogenic factors are reported as mediators related with the tumorigenesis of obesity-related tumors [39, 41]. Recently, it was even shown that higher VFA is associated with a higher incidence of pancreatic cancer and lung squamous cell carcinoma based on epidemiological analyses [42]. However, there is a definite need for further investigations to analyze interactions of visceral fat and tumor micromilieu directly. Importantly, the underlying mechanism of why VFA is linked to OS in patients with gastric cancer is still elusive.

The present meta-analysis has several limitations. First, there are inhomogeneities among the included studies. Possible reasons are different methods estimating fat areas and different composition of the patient samples. This results in relatively large inhomogeneity indices in the present analyses. Second, there is restriction to English language. Third, only few studies investigated all fat areas comprising all values of adipose tissue, namely, VFA, SFA, and VSR. Most studies analyzed only VFA. Forth, most studies were based on patient samples of Asia, and no study was performed in Europe or North America. Therefore, the present results should be translated with care for patients of these continents. There is definite need for studies investigating gastric cancer patients in Europe and North America. Fifth, only data of univariable analyses could be pooled in this analysis. The included papers did not report sufficient results in a multivariable way. This could result in certain confounders as the effect of the CT-defined fat parameters is not adjusted to already established prognostic markers. However, as the present meta-analysis pools data of several published papers, the present results can nevertheless be considered reliable.

## Conclusion

CT-defined VFA and SFA are associated with OS in patients with gastric cancer. VFA influences also the occurrence of postoperative complications. Therefore, assessment of fat areas should be included in clinical routine in patients with gastric cancer.

## Statement of Ethics

An ethics statement is not applicable because this study is based exclusively on published literature.

## Conflict of Interest Statement

All the authors have indicated that they have no conflict of interest to declare.

## Funding Sources

The authors did not receive any funding.

## Author Contributions

All the authors have contributed equally to this work. Hans-Jonas Meyer drafted the manuscript. Alexey Surov designed and supervised the study. Andreas Wienke performed the statistical analysis. Maciej Pech designed and supervised the study. All the authors discussed the results and approved the final manuscript for publication.

## Data Availability Statement

All data generated or analyzed during this study are included in this article. Further inquiries can be directed to the corresponding author.

## Supplementary Material

Supplementary data

## Figures and Tables

**Table 1:** Overview of the included studies

Authors	Country	Study design	Included patients, *n*	Diagnosis, tumor stage	Treatment	Mean age, years	Gender, female, *n* (%)	Patients with high fat area, *n* (%)	Definition of fat area	Calculation of fat area	Defined Hounsfield Units for fat area	Outcome
Bitencourt et al., 2019 [12]	Brazil	Retrospective	70	52 gastric cancer, 18 esophageal-gastric junction tumors, unclear tumor stage	54 (77.1%) with neoadjuvant treatment	59.9	23 (32.9)	41 (48.0)	Above 100 cm^2^	VFA and SFA on L4/5 level	−190 to −30	OS
Carvalho et al., 2021 [13]	Brazil	Prospective	84	Partial gastric cancers (*n* = 29, 34.5%); 18 I, 21 II, 26 III, 16 IV, and 3 unknown	; Gastrectomy, not specified	59.7	48 (57.1)	37 (44.0)	163.8 cm^2^ for men and 80.1 cm^2^ for women	VFA on L3 level	−150 to −50	Postoperative complications
Choi et al., 2018 [14]	South Korea	Retrospective	96	Mucosal gastric cancer	Endoscopic resection and surgery	63.5	30 (31.3)	Not stated	Above 100 cm^2^	VFA and SFA on L3 level	−150 to −50	OS, DFS
Dong et al., 2020 [15]	China	Prospective	1,147	437 I, 273 II, 437 III	724 patients with subtotal gastrectomy, 423 total gastrectomy	65	299 (26.1)	616 (53.7)	Above 100 cm^2^	VFA and SFA on L3 level, VSR	−150 to −50	OS, DFS
Huang et al., 2020 [16]	China	Prospective	597	215 I, 157 II, 225 III	Radical gastrectomy	72	134 (22.5)	Not stated	Above 100 cm^2^	VFA and SFA on L3 Level, VSR	−150 to −50	OS and DFS
Park et al., 2018 [17]	South Korea	Prospective	136	57 II, 79 III	D2 gastrectomy with adjuvant capecitabine and oxaliplatin	55	40 (29.4)	67 (47.9)	Over 40.8 cm^2^/m^2^	VFI, SFI on L3 level	−150 to −30	DFS
Taniguchi et al., 2020 [18]	Japan	Retrospective	567	376 I, 99 II, 90 III, 2 IV	Radical gastrectomy	67	174 (30.7)	208 (36.7)	Above 100 cm^2^	VFA on umbilical level	Not reported	Postoperative complications
Wang et al., 2017 [19]	China	Prospective	859	239 I, 193 II, 427 III	Radical gastrectomy	64	187 (21.8)	308 (35.9)	Above 100 cm^2^	VFA on L3 level	−150 to −50	OS and DFS
Zhang et al., 2018 [20]	China	Prospective	157	48 I, 27 II, 81 III	111 subtotal, 45 total gastrectomy; neoadjuvant chemotherapy was administered to 35 patients (22.4%)	59.1	41 (26.3)	64 (41)	VSR over 1.33 in men and over 0.93 in women	VFI, SFI on L3 level, VSR	−150 to −50	Postoperative complications

VFA, visceral fat area; SFA, subcutaneous fat area; VSR, VFA to SFA ratio; VFI, visceral fat index; SFI, subcutaneous fat index; OS, overall survival; DFS, disease-free survival.

**Table 2:** Quality of the studies assessed by NOS scale. The asterisk expresses a positive evaluation of the category for the study

Study	Is the case definition adequate?	Representativeness of the cases	Selection of controls	Definition of controls	Comparability of cases and controls on the basis of the design or analysis	Ascertainment of exposure	Same method of ascertainment for cases and controls	Nonresponse Quality rate score
Bitencourt et al., 2019 [12]		*	*	*	*	*	*	*	7
Carvalho et al., 2021 [13]	*	*	*	*	*	*	*	*	8
Choi et al., 2018 [14]		*	*	*	*	*	*	*	7
Dong et al., 2020 [15]	*	*	*	*	**	*	*	*	9
Huang et al., 2020 [16]	*	*	*	*	**	*	*	*	9
Park et al., 2018 [17]		*	*	*	*	*	*	*	7
Taniguchi et al., 2020 [18]	*	*	*	*	*	*	*	*	8
Wang et al., 2017 [19]	*	*	*	*	*	*	*	*	8
Zhang et al., 2018 [20]	*	*	*	*	*	*	*	*	8

The asterisk expresses a positive evaluation of the category for the study. NOS, Newcastle-Ottawa Scale.
